# Studying Scripts of Women, Men and Suicide: Qualitative-Method Development and Findings from Nepal

**DOI:** 10.3390/ijerph20116032

**Published:** 2023-06-01

**Authors:** Silvia Sara Canetto, Andrew D. Menger-Ogle, Usha Kiran Subba

**Affiliations:** 1 Department of Psychology, Colorado State University, Fort Collins, CO 80523, USA; 2 Department of Psychology, Trichandra College, Kathmandu 44600, Nepal

**Keywords:** suicide scripts, suicide beliefs, lay theories, women, men, Nepal, qualitative method, suicide causes, suicide prevention

## Abstract

Information about suicidal behavior in Nepal is limited. According to official records, suicide rates were high until the year 2000 and declined thereafter. Official records are considered unreliable and a gross undercounting of suicide cases, particularly female cases. Suicide research in Nepal has been mostly epidemiologic and hospital-based. Little is known about how suicide is understood by Nepali people in general—including dominant suicide attitudes and beliefs in Nepal. Suicide attitudes and beliefs, which are elements of a culture’s suicide scripts, predict actual suicidality. Drawing on suicide-script theory, we developed and used a semi-structured survey to explore Nepali beliefs about female and male suicide. The informants were adult (*M_age_* = 28.4) university students (59% male). Female suicide was believed to be a response to the society-sanctioned oppression and abuse that women are subjected to, in their family and community. The prevention of female suicide was viewed as requiring dismantling ideologies, institutions, and customs (e.g., child marriage, dowry) that are oppressive to women, and ensuring that women are protected from violence and have equal social and economic rights and opportunities. Male suicide was believed to be a symptom of societal problems (e.g., unemployment) and of men’s psychological problems (e.g., their difficulties in managing emotions). The prevention of male suicide was viewed as requiring both societal (e.g., employment opportunities) and individual remedies (e.g., psychological counseling). This study’s findings suggest that a semi-structured survey can be a fruitful method to access the suicide scripts of cultures about which there is limited research.

## 1. Introduction

Information about fatal and nonfatal suicidal behavior in Nepal is limited and of questionable reliability. According to data published by the World Health Organization (WHO) in 2014 and 2021, suicide rates in Nepal have varied widely over time [1,2]. According to the 2014 WHO report, in 2000 and 2012 Nepal had among the highest female mortality by suicide in the world. Age-standardized female suicide rates were estimated at 27.1 per 100,000 in 2000, and 20 per 100,000 in 2012. Age-standardized male suicide rates were 40.5 per 100,000 in 2000, and 30.1 per 100,000 in 2012. Analyses of suicide rates by age group revealed that in 2000 and 2012 female and male suicide rates were similar in the 15–29 and in the 30–49 age groups. By contrast, the female–male gap in suicide rates was large in the 50–69 age group and even larger in the 60 and older age groups. Consistent with the 2014 WHO report, a Nepali government eight-district study found that in 2008–2009 suicide was the leading cause of death among Nepali women of reproductive age across ethnicities [3]. According to the 2021 WHO report, however, suicide rates in Nepal were substantially lower in both women and men in 2019 [2]. At that time, the female age-standardized suicide rate was 2.9 per 100,000 and the male age-standardized suicide rate was 18.6 per 100,000 [2]. With regard to method, a study of police records found that, during the period between 1980 and 2019, hanging and self-poisoning by pesticide were the most common suicide methods [4].

There are no official estimates of nonfatal suicidal behavior and suicidal ideation rates in Nepal. A school-based study of adolescent students across five regions found that girls were more likely to report suicidal ideation and nonfatal suicidal behavior than boys [5]. An interview study of married women aged 15–24 found that approximately three-quarters had experienced sexual violence within the marriage, with suicidal thoughts being a common response to the violence [6]. A multiple-district study recorded high rates of suicidal ideation among widows, especially during the year following the husband’s death [7].

There are indications that rates of fatal and nonfatal suicidal behavior increased during the COVID-19 pandemic, especially among women [8,9,10].

In Nepal, suicidal behavior, nonfatal and fatal, is to be reported to the police although there is no criminal liability for suicidal behavior. Since 2017, the abetment of suicide has been criminalized in Nepal. The 2017 code was established mostly to prevent husbands and in-laws from driving women to suicide through mental and physical abuse, including unrelenting dowry claims [11,12,13]. An unintended consequence of the new code is that more suicides likely are reported as accidents, if they are reported at all. There is evidence (e.g., [14]) that suicide is less likely to be reported when it involves women. As a result of these social factors, Nepali official records are a gross undercounting of suicide cases, particularly female cases [15,16]. Nepali records of female suicide may also be inaccurate by way of over-inclusion. It has been noted that the murders of women by family members, for example, because of pregnancy while single or because of dowry greed, may be reported as suicides [17]. In light of all of these reporting issues, rates and patterns of women’s and men’s suicidality in Nepal must be interpreted with caution.

### 1.1. Nepali Women’s Suicidal Behavior: Who, How, and Why

Research on suicide in Nepal has predominantly been hospital- or police-record-based, and descriptive. Several studies did not separately examine female and male suicide patterns [5,18,19].

The hospital- and police-based descriptive studies that analyzed patterns by sex indicated that Nepali women who are suicidal and/or die of suicide are typically young and married (see [20] for a review). A psychological autopsy study of police cases, with the majority (28 out of 39) of informants being male relatives or friends, found that half of the female decedents had children; and that hanging (61.1%) and poisoning (33.3%) were common suicide methods [21]. Mental disorders were reported to be a factor in female nonfatal and fatal suicidality in one-third of the studies reviewed by Kasaju and colleagues [20]. With regard to causes of female suicide, discrimination, abuse, and a culture mandating female subservience and silence have been considered most relevant. For example, in a review of studies by Marahatta and colleagues [22] female suicide was explained in terms of “social hardship… such as poor empowerment of women, lack of educational opportunities… cultural norms restricting self-expression, space and choice,… [and] child marriage” (p. 46). Experiencing violence from an intimate male partner and having no recourse against it has also been linked to female suicide in Nepal [3].

A qualitative analysis of interviews with mostly male relatives or friends of persons who died of suicide (cases that were identified via police records) generated three narratives of female suicide [23]. One narrative was that the women took their life because of shame after having engaged in a behavior (e.g., “a forbidden love relationship”) that “damaged… [their] *ijjat* (social status) … in the community, devaluing… [their] worth and purity…. [and] the *ijjat* of the family”. In this narrative “[s]hameful situations for women typically placed individual blame or fault directly on her” (p. 719). Another narrative was that the suicide was a response to chronic abuse by the husband or male partner, in-laws, paternal family, and/or community. In many of these cases, alcohol abuse by the husband was an additional stress experienced by the women who took their lives. The informants believed that the women had been driven to suicide by the fact that, in their community, women have no recourse against the abuse that they suffer. Therefore, these female suicides were framed as indirectly perpetrated homicides. The third narrative was that the women’s deaths were covered-up homicides perpetrated by their family. Taken together, the findings of this study indicate that people saw the suicide of their female relative or friend as a response to family- and community-condoned oppression and abuse.

### 1.2. Nepali Men’s Suicidal Behavior: Who, How, and Why

Information about men who are suicidal and/or die of suicide in Nepal, as separate from information about women, is often unavailable in hospital- and police-based descriptive studies (see [14] for a review). A psychological autopsy study [21] that examined police records by sex found that most of the men who died of suicide were married (81%) and had children (76.2%). Hanging (66.7%) and poisoning (23.8%) were these men’s most common methods. About one-third of men had been involved in migrant labor. The same study found that on the day of the suicide about half of the men had consumed alcohol. None was reported to have a mental illness at the time of the suicide. In a study by Pradhan and colleagues [3], occupational issues such as unemployment were reported to be relevant to male suicide.

A qualitative analysis of interviews with mostly male relatives or friends of persons who died of suicide generated three narratives of male suicide [23]. One narrative was that the men took their life because of shame. Male shame suicide was connected to “broad situations… characterized as being out of the man’s immediate control (e.g., crops failing, general situations of poverty)” rather than a specific event (p. 719). A second narrative was that alcohol abuse was to blame for the suicide and its antecedents, including poverty and the deceased man’s abusive behavior towards his family. A third narrative was that the suicide was a response to financial problems. This narrative was more common in the case of younger men’s suicides. These “[m]en were described as feeling like a failure because of financial burdens or an inability to succeed economically”. It was stated that “[t]his sort of failure in males often led to alcohol abuse, perpetuating feeling of shame and damage of one’s *ijjat*” (p. 719). Difficulties re-integrating in the family following migrant work was a thread across the three narratives.

### 1.3. Strengths and Limitations of Past Studies of Female and Male Suicide in Nepal

Hospital and psychological autopsy studies have provided information on patterns and themes of female and male suicide in Nepal in terms of the characteristics of the suicide decedents, and the methods they use, and in terms of possible suicide explanations. In this way, the hospital and psychological autopsy studies have started making visible the scripts of female and male suicide in Nepal.

With regard to suicide explanations, a limitation of hospital studies is that they record the perspectives of professional staff. Professionals’ views of suicide are influenced by their training and by the interests and politics of their profession. A limitation of psychological autopsy studies is that the suicide explanations of family and friends are shaped by the relationship the informants had with the deceased and by the emotional and social impact that the suicide had on them.

An important way to access the suicide scripts of a culture is via studies of the suicide beliefs of so-called lay people, that is, people who are not professionally involved with suicide [24,25,26,27,28,29,30,31,32,33,34,35,36,37,38]. Because suicide is a relatively rare event, most lay persons have not experienced a death by suicide in their close relationships—though someone close to them may have had suicidal thoughts. Studies of lay persons’ beliefs about suicide provide a unique window into a culture’s scripts of suicide.

### 1.4. Cultural Scripts of Suicide: Theory and Findings

A culture’s suicide scripts include the conditions under which suicidal behavior is relatively acceptable, and even expected in that culture—by whom, with what method, when, why, and with what consequences [39,40,41,42]. A culture’s suicide scripts are both descriptive and prescriptive. A diversity of evidence shows that a culture’ suicide scripts influence the likelihood of suicidal behavior in that culture [43,44,45,46,47]. For example, a U.S. longitudinal study found that suicide acceptability (an element of suicide scripts) predicted suicide in the general population—by a twofold increase, in some cases [45]. A culture’ suicide scripts also influence the form that the suicidal act takes (e.g., the suicide method and the circumstances of the suicide) and the suicidal act’s outcome (see [42] for a review).

Research on suicide scripts has used both quantitative (e.g., [30,36,48,49]) and qualitative methods. A qualitative approach is most common and appropriate when there is limited information about the suicide scripts of a culture or social group [24,25,26,27,28,29,31,32,33,34,37,38,50,51,52,53,54,55,56,57,58,59,60,61]. As argued by Staples and Widger, to understand suicidality, we need to learn “how suicidal behaviours are imagined, talked about, and practiced; how they relate to other kinds of behaviours and other kinds of institutions; when and under what possibilities different people in the communities we study think suicide might arise and when it might not, when it might be ‘acceptable’ and when it might not; and how suicidal behaviour does not begin with the ‘precipitating factor’ and end with the ‘suicidal act,’ but extends deep into individual and collective pasts and futures” [62] (p. 199).

### 1.5. This Study

In the current study, we developed and used a semi-structured survey to explore female and male suicide scripts in Nepal. Drawing on suicide-script theory [39,40,42] and research [28,29,31,33,36,55,63], we documented views of the “who” (i.e., beliefs about the typical age and civil status of women and men who die of suicide) and “how” of suicide (i.e., beliefs about the methods used by women and men who die of suicide, and why they use those methods), as well as views of warning signs (i.e., behaviors believed to suggest that a woman or a man is considering suicide). In addition, we recorded views of the “why” of suicide (i.e., beliefs about the causes of female suicide versus the causes of male suicide) and the “why not” of suicide (i.e., views about what could prevent female suicide and what could prevent male suicide). Finally, we requested participants to provide a definition of suicide and to give us feedback about the survey.

## 2. Methods

### 2.1. Sample

This study’s informants were 74 Nepali adults (58% male, *M_age_* = 28.4; 51% never married). At the time of data collection, they were graduate students at Trichandra College, an institution which is part of Tribhuvan University, Kathmandu. The participants’ main fields of study were Sociology (24%), Rural Development (18%), Psychology (8%) Education (7%), and Management (7%); 18% of participants were in other fields of studies and 19% did not report their field of study. The participants had lived in Kathmandu for a median of nine years.

### 2.2. Recruitment and Procedure

Recruitment and data collection were structured based on recommendations of Nepali professionals, including a psychology professor and a mental-health therapist. The study was advertised among graduate students in a program that requires English-language fluency. Data collection was conducted in classrooms at Trichandra College. During data collection sessions, a Nepali research assistant read a description of the study and the informed-consent statement and then distributed paper versions of the informed-consent statement. No participation incentives were offered to the respondents. After the participants read and signed the informed consent, they were given access to a paper copy of the survey. Participants had between 20 and 30 min to complete the survey. After they finished, participants were given a written debriefing statement that included information about counseling services that they could access, should they wish to discuss issues and feelings triggered by the survey. Data collection was conducted in 2014.

### 2.3. Measures

A survey including a combination of Likert-scale and sentence-completion items was used to probe key elements of suicide scripts. The theoretical foundation of the survey is suicide-script theory [39,41,42].

The survey was developed by the study’s authors. Each author brought unique expertise to the project. Canetto is a bi-national (Italy/USA) counseling, clinical, social, and lifespan psychology professor at Colorado State University, USA, and a suicide-scripts scholar. Menger-Ogle is a U.S.-born social psychologist who completed his doctorate at Colorado State University. Menger-Ogle spent 6 months in Nepal where he collaborated with a diversity of local professionals on refining this study’s method and then collecting this study’s data. Subba is a Nepal-born and -educated psychology professor, currently at Trichandra College, Nepal. The study’s survey development benefited from feedback from a Nepali mental health therapist and a Nepali research assistant. Prior to being used with this study’s sample, the survey was tested with two groups (n = 6; and n = 11) of Nepali university students, and revised based on these students’ feedback. The informed consent and survey were in English because the target sample was graduate students in a program that requires English-language proficiency.

There were two versions of the survey. One focused on female suicide and the other on male suicide. Thirty-six informants (61% male) responded to questions about female suicide and thirty-eight (55% male) responded to questions about male suicide.

The first set of survey items asked respondents to indicate how knowledgeable they were about suicide in Nepal (on a scale of 1–5), how they had learned about suicide in Nepal (based on 5 options); and how big of a problem they thought suicide is in Nepal (on a scale of 1–5). The respondents were also asked to indicate whether suicide is more of a problem for women than for men, vice versa, or equally for women and for men.

The next set of questions requested that participants indicate what they thought are the typical age and marital status of women (or men) who kill themselves, and what the participants believed are women’s (or men’s) typical suicide methods and the reasons for the chosen methods.

Participants were then asked to describe a common warning sign of suicide in Nepali women or a common warning sign of suicide in Nepali men.

Following that, participants were asked to indicate why Nepali women (or Nepali men) kill themselves and what could prevent women’s (or men’s) suicide.

The last section of the survey requested feedback about the survey (i.e., clarity of the survey on a 1–5 scale; items that participants found difficult to understand and why, and recommendations about questions to be included in future studies of suicide in Nepal). The final survey question asked for a definition of suicide (see the Appendix A for the full text of the suicide-script survey).

### 2.4. Data Analyses

Braun and Clarke’s [64] thematic-analysis method was used to code and organize into themes the responses to the sentence-completion questions. A multi-national research team led by Menger-Ogle processed the suicide-scripts data. Menger-Ogle and four research assistants (a Bangladeshi woman, a Romanian woman, a U.S. woman, and a U.S. man) coded the responses. Canetto and Subba served as consultants to the data analysis. A semantic approach was taken to the sentence-completion responses. Team members familiarized themselves with the data, generated initial codes, searched for patterns within the explicit meaning of the data, reviewed the patterns, and then defined and named the themes represented by the patterns. The process through these phases was iterative, not linear. Toward the end of the data-analyses process, quotes were chosen to provide examples of the themes. Information about specific topics (e.g., suicide prevention) was not necessarily or only given in response to those topics’ questions (e.g., the questions on prevention). Therefore, coding for specific content drew across the full data set. Responses regarding feedback about the survey and the suicide-definition data were processed by the study’s authors.

## 3. Results

### 3.1. General Views of Suicide in Nepal

#### 3.1.1. Quantity and Quality of the Data

There were 210 responses to the sentence-completion items about female suicide; 189 were comprehensible and usable. Of the 228 responses to the sentence-completion items about male suicide, 216 were usable.

Participants rated the survey as moderately clear (*M =* 3.4, *SD* = 1.0, for the female survey; *M* = 3.6, *SD* = 1.2, for the male survey). The item that participants found most difficult to understand was the question about warning signs of suicide.

#### 3.1.2. Participant’s Views of Their Knowledge about Suicide in Nepal

The respondents believed that they had adequate knowledge about suicide (*M =* 2.8, *SD* = 1.0, for the female version of the survey; *M* = 2.9, *SD* = 1.0, for the male version). Most reported having learned about suicide because suicide had happened in their community (29% in the female version of the survey; 38% in the male version); or via media stories (39% in the female version of the survey; 35% in the male version). No participant learned about suicide through a job that dealt with suicide.

#### 3.1.3. How Big of a Problem Is Suicide in Nepal?

Participants who responded to the questions about female suicide believed that suicide is a moderately serious (*M* = 3.2, *SD* = 1.2) problem in Nepal. The participants who expressed views about male suicide also believed that suicide is a moderately serious (*M* = 3.6, *SD* = 1.2) problem in Nepal.

#### 3.1.4. Who Do You Think Suicide Is Mostly a Problem for in Nepal?

Most (66%) respondents reported a belief that suicide is mostly a problem for women or a problem for women and men equally (30%). Only four percent believed that suicide is mostly a problem for men in Nepal.

#### 3.1.5. How Do You Define Suicide?

In response to this question, respondents provided a description of suicide (e.g., *killing oneself* and *the act of intentionally causing one’s death* wrote a female and a male respondent, respectively). Respondents also wrote about the social (*the society and state are responsible* wrote a male participant) and/or the psychological problems (*to get rid… from the pain of living* stated a male participant) that they believed trigger suicide, and how these social and psychological problems may be linked (*negative thinking… cause* [sic] *by social cultural and economical disorder of Nepalese society toward women* wrote a male respondent). There were comments about suicide as a foolish person’s act (*person seem* [sic] *to be foolish* wrote a male respondent), an illness (*disease* wrote a female respondent), a mental disorder (*a mental disorder which forces one to kill himself* wrote a female respondent) or a way to achieve a new soul (*a person thinks this world is full of sin and troubles and wants to be free from his soul to achieve a new one* stated a male participant).

### 3.2. Views of Female Suicide

#### 3.2.1. What Kind of Nepali Woman Is Mostly Likely to Suicide?

Most participants (76%) believed that women who die of suicide typically are ages 20 to 24. Half of participants expressed the view that suicide is more common among married women and the other half thought that it is more common among single women.

#### 3.2.2. Using What Method, Typically, and Why?

The respondents believed that common suicide methods used by women are hanging (78%) and poisoning (73%). Examples of poisoning included medication and pesticides. Other methods mentioned as typical among women were drowning, burning, and jumping from a height. The respondents expressed the view that women use these methods because they are widely available, easy to execute, effective, and discrete.

#### 3.2.3. What Are Warning Signs of Suicide for Nepali Women?

The respondents believed that warning signs of suicide for Nepali women are isolating oneself (*stays alone* wrote a female respondent) and negative emotions (*frustration, irritation, anger* said a female respondent; *depression and stress* and *depression due to love and social problems* said two male respondents, respectively). Having family problems (*suffering from violence* wrote a female respondent) was also mentioned as a warning sign of female suicide.

#### 3.2.4. Why Do Nepali Women Kill Themselves?

Statements about the perceived causes of female suicide in Nepal were classified into three categories: individual, interpersonal, and societal.

Female suicide was linked to negative emotions (*they are in frustration* wrote a female respondent; *depression* stated several male respondents; *when they are highly discouraged, frustrated, when they face the embarrassing moment*… [that] *they couldn’t tolerate* wrote a male respondent) and negative thinking (*helpless* stated a female respondent; *They think they have no way to go forward* wrote a female respondent).

Female suicide was also associated with interpersonal problems. When the interpersonal problems were named, they were described as physical and mental abuse and oppression perpetrated by family members, usually after marriage *(physical and mental violence* stated a female respondent; *regular repetition of domination (physically and mentally) done to them* stated a male respondent). Disappointment and failure in love relationships were also presumed causes of female suicide (*if they have a failure* [in] *their love especially teenagers* wrote a female respondent; *break up in their love* and *they fail in love and or their marriage fails* wrote two male respondents, respectively).

Finally, the societal causes of female suicide were thought to be the oppression and discrimination that women experience in their community and the oppression, abuse, and violence that women are subjected to in their family (all without a possibility for recourse), combined with the inadequate economic resources and the limited work and life opportunities that women have in Nepal (s*ocial abuse* wrote a male respondent; s*ocial structure, norms and values, discrimination* wrote a female respondent*; harassment, domination, poverty* wrote a male respondent; *unemployment* stated another male respondent). The dowry system (*dowry system* and *pressure for dowry* wrote two female respondents, respectively) was also mentioned a societal factor in women’s suicide.

#### 3.2.5. What Could Prevent Nepali Women’s Suicide?

Suicide-prevention ideas were classified into three categories: individual, interpersonal, and societal.

At the individual level, respondents thought that female suicide could be prevented if women had more psychological resources, including a stronger sense of their value and importance (*feel about their importance in family, society and country* wrote a female respondent). Participants also believed that the prevention of female suicide requires that women develop their coping skills and psychological stamina so they can better sustain the challenges of the fight for social and economic rights (*learn about different skills and get good psychological counseling* wrote a female respondent; *awareness on rights* said another female respondent).

Family and friends were believed to have a role in reducing female suicide. Respondents said that family members should stop abusing their female relatives (*not involving them in the physical harassments* wrote a male respondent). Respondents also noted as critical that families be educated about the discrimination and abuse that women experience in Nepali society. Emotional support, love, appreciation, respect, and affirmation were mentioned as family’s and friends’ necessary contributions to the prevention of female suicide (*give proper support and encourage them* and *give value to their wants* wrote two male respondents, respectively).

In terms of societal suicide-prevention initiatives, female suicide was believed to be preventable via ending the discrimination, oppression, and abuse that women experience in the family and in society (*end of male domination, elimination of social discrimination,* and *empower women politically, economically, socially and treat them equally* wrote three male respondents, respectively; *freedom* and *empowered* wrote two female respondents, respectively). Ensuring that women have social, educational, employment, and economic opportunities equal to those of men was also listed as a societal responsibility in the prevention of female suicide (*equal opportunity* wrote a female respondent, and *be economically independent and well educated* wrote a male respondent). The government was viewed as having a role in providing education about the role that the abuse of women’s rights has in female suicide (*bringing awareness about the rights of women* wrote a male respondent). Enacting laws to punish male domination and male violence against women was also recommended as a societal measure to prevent female suicide (*make strict law for the family member who use to dominate their female member* and *could implement strict laws for crimes like rape, violence* wrote two male respondents, respectively).

### 3.3. Views of Men’s Suicide

#### 3.3.1. What Kind of Nepali Man Is Mostly Likely to Suicide?

Most participants (61%) thought that men who die of suicide typically are ages 20 to 24. About half (45%) endorsed the idea that suicide is more common among married men and about half (45%) thought that suicide is more common among single men.

#### 3.3.2. Using What Typical Method and Why?

The respondents believed that the suicide methods typically used by men are hanging (71%) and poisoning (61%). Other methods thought to be common among men were drowning, burning, and jumping from a height. The respondents expressed the belief that men use these methods because they are widely available, easy to execute, effective, and discrete.

#### 3.3.3. What Are Warning Signs of Suicide for Nepali Men?

The respondents believed that warning signs of suicide for Nepali men are isolating oneself (*stay alone, not talking to anyone* said a female respondent), withdrawing from responsibilities *(ignoring family & work* said a female respondent) and negative emotions (*anger, sad,* wrote two male respondents; *frustration, depression* wrote several female respondents).

#### 3.3.4. Why Do Nepali Men Kill Themselves?

Statements about the perceived causes of male suicide in Nepal were classified into three categories: individual, interpersonal, and societal.

Male suicide was associated with negative emotions (*they are frustrated* said several female and male respondents; *depression due to family problems, pressure of education by the family, love affairs* wrote a female respondent), negative thinking (*they feel they cannot solve that problems* [sic] wrote a male respondent) and reduced psychological stamina *(less capacity of bearing problems* wrote a male respondent; *they cannot control their emotion* wrote another male respondent). Failures (*unsuccess* said two female respondents; *failure in their love* said a male respondent; *fail in exams or fail in love affairs* said a female respondent) were also mentioned as individual causes of male suicide.

Male suicide was also linked to interpersonal problems. When these problems were named, they were described as family problems (*family problems* and *home problems and misunderstanding* wrote two male respondents, respectively; *feels neglected by his family or by friends* wrote a male respondent). Problems in love relationships (*failure in their love* stated a male respondent) were also presumed causes of male suicide.

Socioeconomic problems, including lack of education, lack of jobs or poorly paid jobs, poverty, and economic crises, were mentioned as societal causes of male suicide *(economic crisis, low income* and *their economical* [sic] *base is very crisisful* [sic] stated two male respondents, respectively; *unemployment* wrote a female respondent; it is because *they don’t support family and society* wrote another female respondent).

#### 3.3.5. What Could Prevent Nepali Men’s Suicide?

Suicide-prevention ideas were classified into three categories: individual, interpersonal, and societal.

At the individual level, respondents thought that male suicide could be prevented if men changed their behavior (*share their feelings openly* and *control their sentiments* wrote a female and a male respondent, respectively). Participants also believed that the prevention of male suicide requires that men have psychological support, including via access to psychological counseling (*psychological treatment and good counseling* wrote a female respondent; *counseling for promoting self actualization enhancing esteem* wrote a male respondent). Education about suicide (*awareness program, increase in education* wrote a male respondent; *information through media*, wrote a female respondent) was mentioned as another individual-focused way for the prevention of male suicide

Family and friends were believed to have a role in reducing male suicide. Family’s and friends’ emotional support, understanding, care, love, and respect were viewed as critical to the prevention of male suicide (*proper care and love* and *could understand their problem and support them* wrote two female respondents, respectively; *help them in a good way* wrote a male respondent).

In terms of societal suicide-prevention initiatives, male suicide was believed to be preventable by increasing men’s employment opportunities (*employment* wrote a male respondent; *opportunities to work with a good salary to complete family needs* wrote a female respondent; *get job in Nepal* wrote a male respondent). The government was seen as having a role in increasing men’s awareness and education about suicide (*give information through media* and *do awareness campaign* [sic] *in village/city* said two female respondents, respectively; *topic in lesson at college curriculum, give education about the value of life, promote psychological counseling*, and *classes about psychological counseling in primary… school* wrote four male respondents, respectively. It was also suggested that the government enact laws against suicide (*make a strict law against suicide* wrote a female respondent).

## 4. Discussion

This article describes the method that we developed and used to explore beliefs about female and male suicide among Nepali adults, as well as the findings generated using our method. Via our semi-structured survey, we accessed the views of adults in post-graduate programs regarding who is most at risk of suicide in Nepal. We also examined their beliefs about the methods women and men typically use and why; their beliefs about the warning signs of suicide, in women and in men; and their beliefs about what causes and what could prevent women’s and men’s suicide.

### 4.1. Scripts of Female Suicide

A key finding of this study is that female suicide was viewed as having societal causes and as requiring societal remedies.

Specifically, female suicide was considered a response to the society-sanctioned discrimination, oppression, and abuse that Nepali women are subjected to, in their family and community. The narrative that emerged was that women resort to suicide because they have no recourse against the society-sanctioned discrimination, oppression, and abuse. Negative emotions and thoughts, including frustration, depression, and helplessness, were viewed as an understandable response to such discrimination, oppression, and abuse—not as independent drivers of female suicide

Female suicide was believed to require societal solutions—in fact, a social transformation. Specifically, the prevention of female suicide was viewed as necessitating the end of the discrimination, oppression, and abuse that women are subjected to, at the societal and at the family level; and ensuring that women have social, educational, and economic opportunities equal to those of men.

The beliefs about female suicide expressed by this study’s respondents, that the determinants of female suicide are first and foremost societal, are consistent with the beliefs about female suicide that emerged in a psychological autopsy study of Nepali individuals who lost a relative or friend to suicide [21,23]. The role of social and economic factors in Nepali women’s suicide was also highlighted in a scoping review of mostly hospital-based studies [20]. The authors of the review stated that “patriarchy, gender inequity, poverty and rigid socio-cultural norms” are the “root causes” of female suicide in Nepal (p. 10). Also consistent across Nepali studies is the view that the psychological problems that suicidal women experience are a response to the discrimination, oppression, and abuse that women are subjected to—not primary and independent drivers of suicide.

Taken together, the social-context-focused explanations of female suicide that recur in this and in other Nepali studies [6,7,14,15,18,19,21] stand in contrast to the female suicide explanations that are dominant in the literature produced in high-income countries [37,40]. In high-income countries, suicide in general, and female suicide in particular, tend to be understood as psychological problems, and specifically, as a symptom of mental disorders, both by the general public (e.g., in Norway [54]) and by researchers (see [65] for a review of high-income-country studies of suicide and mental illness). The kind of mental disorders that, in high-income countries, are assumed to be primary and independent drivers of women’s suicidal behavior are mood disorders and borderline personality disorder (see [39,66] for critiques of psychiatric explanations of women’s suicidality).

A likely reason for the difference in theories of female suicide by country and by country’s income level is that female suicide truly has different determinants in Nepal, and more generally, in low-income countries as compared to high-income countries. This is a plausible explanation given the evidence that the same behavior, including women’s suicidality, often has different drivers and different meanings (that is, follows a different script) in different cultures and countries [66,67].

The emphasis in high-income countries on mental illness as the primary driver of suicide, particularly in the case of women, also reflects a theoretical tradition. There are indications that high-income countries’ tradition of viewing female suicide as a symptom of women’s psychological deficiencies and mental disorders [63] has persisted beyond its empirical justification. Studies show that social and economic factors are important to the suicidality of women living in high-income countries [68].

### 4.2. Scripts of Male Suicide

A key finding of this study is that male suicide was viewed as having social and psychological causes, with both social and psychological remedies therefore being necessary for prevention.

Societal factors were believed to contribute to Nepali men’s suicide. Unemployment or poor employment (including migrant work) were considered especially important factors in male suicide. Consistent with this narrative, expanding men’s job opportunities was thought to be critical to the prevention of male suicide. Problems in close relationships, including problems in love relationships, were also viewed as relevant to male suicide.

Psychological factors were also believed to be significant in male suicide. Men who die of suicide were thought to have reduced psychological stamina and problems at managing their emotions. Recommendations for the prevention of male suicide emphasized the importance for men of having psychological counseling.

The beliefs about male suicide that were expressed by this study’s respondents, that the drivers of male suicide are both societal and psychological, are similar to the beliefs about male suicide that were expressed by individuals who lost a relative or friend to suicide, in a psychological autopsy study [23]. A difference is that, in the psychological autopsy study, the informants also thought that alcohol abuse had a major role in their male relative or friend’s suicide.

Taken together, the explanations of male suicide that recur in this and in other Nepali studies [2,21] are in some ways similar to, and in many ways different from, the explanations that are dominant in the literature produced in high-income countries [37,40]. Across low-, middle-, and high-income-country studies, male suicide is assumed to be driven by adversities in public domains, particularly employment adversities. A difference is that in Nepal, male suicide is also associated with close-relationship, love, and family problems. Another difference is that, in Nepal, male suicide is understood as an expression of men’s psychological problems, including their difficulties in managing emotions. Consistent with this view, recommendations for the prevention of male suicide include that men learn about and access counseling to improve their psychological skills.

A likely reason for the difference in theories of male suicide by country and by country’s income level is that male suicide truly has different determinants and different meanings (and follows a different script) in Nepal, and possibly in low-income countries, as compared to high-income countries.

The reluctance in high-income countries to consider psychological and close-relationship factors in male suicide also reflects a theoretical tradition. There are indications that the high-income countries’ tradition of viewing male suicide as mostly or exclusively a symptom of their public-life problems (e.g., unemployment) has endured beyond its empirical support. Studies show that men’s private-life behaviors (including men’s participation in family caregiving [69]) and men’s psychological problems (e.g., [70]) are relevant to the suicidality of men living in high-income countries.

### 4.3. This Study’s Limitations and Strengths, and Directions for Future Research

As a study about a sensitive and complex topic, and the first study of the suicide scripts of Nepali lay persons, this study was cautious in its method, including the kind of people approached for participation and the data-collection setting. By design, the sample were adults in graduate school in Nepal’s capital city. Our reasoning was that educated and urban-living participants would be more comfortable responding to direct psychological questions about suicide. Feasibility also influenced our sample choice. This study’s method choices are not unusual. Studies of lay theories of suicide conducted in other countries also had university-student samples (e.g., [29,30,31,36,55,71]).

We are aware that what made the participants relatively easy to recruit and work with is also a limitation of our study, in terms of the generalizability of the results. In this sense, the contribution of this study is more in terms of method development (e.g., creating and testing a suicide-script survey) than in terms of the study’s findings.

This study’s survey had mostly open-ended questions, consistent with the method used, for example, in studies by Fortune and colleagues [28] and Heled and Read [29]. By asking respondents to describe their suicide beliefs in their own words, this study facilitated the emergence of ideas that were not anticipated by the researchers, thereby reducing the influence of the researchers’ theoretical frameworks. Furthermore, the written format of this study’s survey supported anonymity. At the same time, the open-ended, written format of the survey allowed for variability in the length and quality of the responses. To improve the quality of the data, future research using this study’s method could include a brief follow-up individual interview or a focus group, as a way to verify the meanings of the written responses and to help clarify how the response to one question may relate to the response to another question.

Future directions for suicide-scripts research in Nepal include translating this study’s survey into Nepali languages and administering it to informants representing a diversity of backgrounds (e.g., level and type of education) and locations (e.g., rural and urban). Expanding beyond a highly educated sample would require substantial adjustments of the recruitment and data-collection procedures used in this study (see [72] for reflections on, and recommendations for, qualitative research in a low-income country).

A limitation of this study is that its sample was majority-male. This is not surprising given that this study’s participants were master’s program students. In Nepal, the majority of master’s students are male [73]. Hageman and colleagues’ psychological autopsy studies also had a preponderance of men (72%) among the informants—which the authors explained as the result of men in Nepal being “the public gatekeepers for the family” [23] (p. 711). Given the importance of having a female perspective, particularly in Nepal, where women are expected to be silent [15], future studies of suicide in Nepal should seek to overcome the barriers to women’s participation in research and ensure that at least half of the informants are women.

## 5. Conclusions

Drawing on suicide-script theory, in this study we developed and used a semi-structured survey to explore beliefs about female and male suicide in Nepal. We found that female suicide was thought to be a response to the society-sanctioned discrimination, oppression, and abuse that women are subjected to, in their family and community. The prevention of female suicide was viewed as requiring dismantling the ideologies, institutions, and customs that are oppressive to women and that violate women’s rights, including the right to be free from violence; and ensuring that women have equal social and economic rights and opportunities. Male suicide was believed to be a symptom of societal problems (e.g., unemployment) and of men’s psychological problems. The prevention of male suicide was viewed as requiring both societal (e.g., improved employment opportunities) and individual (e.g., psychological counseling) remedies. The scripts of female and male suicide that emerged from this and other Nepali studies are more different from than similar to the suicide scripts of female and male suicide of high-income, Anglophone countries.

A conclusion, based on this study’s method-development experience and outcomes, is that a semi-structured written survey can be a fruitful way to access the suicide scripts of cultures and social groups about whom there is limited research. A conclusion based on this study’s survey findings is that high-income countries’ suicide theories and suicide-prevention practices may not be relevant, or may only be partially relevant, in low- and middle-income countries.

## Data Availability

In order to protect the privacy of the research participants, the responses to the survey cannot be made available.

## Appendix A

**Views of Women and Suicide in Nepal**

** In this survey we ask for your views and perceptions of suicide in Nepal. We are interested in what you have heard about suicide in Nepal. This is NOT a test of your knowledge, so there are no right or wrong answers.**
1. **How knowledgeable are you about suicide in Nepal?** (circle a number)
NOT AT ALL KNOWLEDGEABLE 1 | 2 | 3 | 4 | 5 VERY KNOWLEDGEABLE
2. **How have you learned about suicide in Nepal?** (circle all that apply)
∘ It has happened in my community
∘ I work in a job that deals with suicide
∘ It was a topic covered in presentations in my school/university
∘ Via news reporting in the media (newspapers, radio, TV, etc.)
∘ Via fiction stories in the media (cinema, TV, books, etc.)
3. **Based on what you have heard, how big of a problem do you think suicide is in Nepal?** (circle a number)
A VERY SMALL PROBLEM 1 | 2 | 3 | 4 | 5 | A VERY BIG PROBLEM
4. **Based on what you have heard, who do you think suicide is mostly a problem for, in Nepal?** (circle one)
Women | Men | Equally so for women and men
**Please complete the following sentences about at what age, how (via what method) andwhy women typically die of suicide in Nepal.**
5. Based on what I have heard, when Nepali women kill themselves, they typically are around the age of __________________________________.
6. Based on what I have heard, when Nepali women kill themselves, they are usually (circle one)
single/never married | married | divorced | widowed
7. Based on what I have heard, when Nepali women kill themselves, they typically use a suicide method such as ____________________________________________________________.
8. Based on what I have heard, Nepali women typically use the above-mentioned method(s) because ________________________________________________________________________.
9. Based on what I have heard, it is possible to know that a Nepali woman is thinking about killing herself if she______________________________________________________________.
10. Based on what I have heard, when Nepali women kill themselves, it is usually because _______________________________________________________________________________.
11. Based on what I have heard, a common warning sign of suicide in Nepali women (for example, a behavior suggesting that she is considering killing herself) is _______________________________________________________________________________.
**Please complete the following sentences about suicide prevention.**
12. Suicide among Nepali women could be prevented if women could _______________________________________________________________________________.
13. Suicide among Nepali women could also be prevented if their family _______________________________________________________________________________.
14. Suicide among Nepali women could be further prevented if the government ________________________________________________________________________________
15. Ultimately, the most important step in preventing suicide among Nepali women is _______________________________________________________________________________.
**In this section we ask for feedback about this survey so we can improve it.**
16. How clear (understandable) is this survey? (circle a number)
NOT AT ALL CLEAR 1 | 2 | 3 | 4 | 5 VERY CLEAR
17. The item or items that are difficult to understand in this survey are items number __________. These items are difficult to understand because __________________________ _______________________________________________________________________________.
18. What additional questions do you think should be asked to better understand suicide in Nepal, specifically women’s suicide in Nepal? _______________________________________________________________________________.
19. Finally, please provide your definition of suicide: Suicide is _______________________________________________________________________________.
**Note:** The male version of the survey was identical to the female version, save for replacing the word “women” with the word “men” in items 5–18.
